# Supplementary material for: Are carbon emissions trading and green financial instruments synergistic? -Comprehensive quantitative research based on content analysis
Source: PLoS One. 2024 Mar 7;19(3):e0298601. doi: 10.1371/journal.pone.0298601 (PMC10919601; doi:10.1371/journal.pone.0298601)
Supplement: S1 Data — (ZIP) [file pone.0298601.s001.zip › Data/地级市Z_F工作报告.zip!/╒π╜¡/└÷╦«╩╨/└┤╫╘╣1⁄2╓┌║┼í╛┬φ┐╦╩2╛▌═°í┐_2016.docx]

各位代表：

　　现在，我代表市人民政府向大会作政府工作报告，请予审议，并请市政协委员和其他列席人员提出意见。

　　一、2015年工作回顾和“十二五”发展成就

　　2015年是“十二五”收官之年，也是丽水经济社会发展很不平凡的一年。在省委、省政府和市委的坚强领导下，全市上下齐心协力、克难奋进，坚定不移地推进“绿水青山就是金山银山”战略指导思想落地生根，努力实现生态、经济、社会效益有机统一，较好地完成了市三届人大五次会议确定的目标任务。

　　一是生态为先抓示范。全力打造生态环境保护和生态经济发展“双示范区”，我市成为首批国家级生态保护与建设示范区，缙云创成国家级生态县，松阳、景宁通过国家级生态县技术评估，丽水开发区成为国家级循环化改造示范试点园区。组织编制环境功能区划，将95.8%的市域面积划入自然生态红线区、生态功能保障区、农产品环境保障区。组织开展绿色生态发展“全员轮训”。全面推进“五水共治”、“五气共治”，我市治水经验入选中国基层治水十大经验之一，龙泉、庆元获得全省“五水共治”“大禹鼎”，4个县(市)创成省级“清三河”达标县，全市地表水环境功能区Ⅰ-Ⅲ类水质达标率达98.96%，饮用水水源地水质达标率、跨行政区域河流交接断面水质达标率均为100%；累计淘汰黄标车12917辆，淘汰改造燃煤锅炉726台，市区环境空气质量优良率达90.1%，6个县(市、区)环境空气质量达到国家二级标准。落实生态经济发展长效机制，设立生态经济产业基金，整治“低小散”企业(作坊)1690家，淘汰落后产能涉及企业224家，否决不符合生态环保要求投资近150亿元，与此同时经济运行总体平稳、结构不断优化。据初步测算，实现地区生产总值1102.34亿元，增长6.4%；一般公共预算收入94.51亿元，可比增长8.9%；服务业增加值占GDP比重达到46.1%。

　　二是产业为基抓提升。生态精品农业“361”工程深入实施，“丽水山耕”品牌影响力进一步提升，启动创建省级现代生态循环农业示范市和农产品质量安全示范市，农产品质量安全例行抽检综合合格率全省第一，遂昌成为国家现代农业示范区。生态工业加快转型，实施“机器换人”示范项目70个，绿谷信息产业园新增入园企业50家，开展首批20家市级“三名”企业培育试点；新设企业增幅居全省第二，新增“新三板”挂牌企业10家、规上工业企业98家，富来森中竹科技有限公司在美国纳斯达克挂牌上市；丽水智能装备及机器人、龙泉汽车空调零部件产业基地成为国家火炬特色产业基地，龙泉、缙云工业园区升格为省级经济开发区。生态服务业加快发展，服务业投资增长14.8%，尤其是生态旅游业带动作用进一步凸显，全年旅游总收入426.02亿元、增长25.5%，九龙湿地创成国家湿地公园。优化电子商务发展政策，促进508家规模企业开展电子商务应用，建成2200多家村级电子商务服务站，实现网络零售额121.43亿元，增长76.0%，网络营销顺差20.49亿元。外贸出口增长19.2%，社会消费品零售总额增长9.0%。

　　三是项目为重抓推进。围绕扩大有效投资抓项目，引进大项目125个，其中5亿元以上大项目22个，实际利用内资293.33亿元、外资2.19亿美元，浙商回归到位资金147.87亿元，固定资产投资增长13.0%。通过“百日攻坚”等行动，重点建设项目完成年度投资计划的120.2%，金丽温高速铁路建成并投入运营，51省道龙泉段改建工程等一批重大项目建成，衢宁铁路丽水段全线开工。特色小镇成为扩大有效投资重要平台，全市谋划建设特色小镇39个，完成投资165亿元，其中8个特色小镇入选省级创建名单，4个列入省级培育名单。推进城市“六个一”示范项目建设，全市新增城市道路44.7公里、城市公园绿地86.99公顷，新开工地下空间70.96万平方米，完成中心城市和美丽县城建设投资74.39亿元。成功承办全省美丽县城暨村庄规划设计现场会。“六边三化三美”工作机制获“2015中国全面小康十大社会治理创新奖”，完成沿线房屋外立面改造366个村5.3万户1707万平方米，建成沿线产业景观带项目237个8.8万亩。

　　四是改革为要抓突破。扎实推进国家生态文明先行示范区的“先行先试”，探索建立生态环境损害责任追究制度，开展自然资源资产责任审计试点，深入推进排污权有偿使用和交易试点。深化农村金融、扶贫、低丘缓坡开发利用等全国改革试点，新增“三权”抵押贷款19.6亿元，龙泉、青田、缙云成为全国“两权”抵押贷款试点县(市)，全面消除家庭人均年收入低于4600元的贫困现象，全市低丘缓坡试点区块基本具备净地出让条件。围绕培育生态旅游第一战略支柱产业，推进旅游体制机制改革，我市成为省级旅游综合改革试验区，市、县两级均完成旅游委员会组建工作。围绕正确处理政府、市场与社会的关系，推进重要领域改革，建立全省首个审批中介服务网上竞价系统，探索企业“零地”投资项目政府不再审批和企业独立选址项目高效审批，我市成为全省政府和社会资本合作示范市、各类事业单位统一登记管理试点市。

　　五是民生为本抓保障。民生大事实事总体上顺利推进，全市民生类支出达210.96亿元，增长18.4%，占一般公共预算支出的75.5%。城乡居民人均可支配收入增速快于经济增速，分别增长8.1%、10.0%，达到32875元、15000元，农民收入增幅连续7年居全省第一。职工最低工资标准平均提高13%，企业退休人员基本养老金标准平均提高10%，城乡居民基础养老金最低标准提高20%，城乡最低生活保障标准分别提高10.2%、18.4%。实施全民参保登记。完善城乡医疗保障，大病保险最高报销限额提高到20万元，报销比例提高到55%。被征地农民基本生活保障制度与职工基本养老保险制度实现并轨。新竣工保障性安居工程住房3809套，完成农村困难群众危旧房改造3100户。高考纯文理第一批上线1676人，总上线率达到省平均水平，丽水五中建成并投入使用，松阳成为我市首个省级基本实现教育现代化县。优质医疗资源“双下沉、两提升”和分级诊疗工作扎实推进，启动人口健康信息化项目。成功创建国家卫生城市。“丽水乡村春晚”获得国家公共文化服务体系示范项目创建资格，我市成为“中国十佳运动休闲城市”，丽水博物馆和美术馆开馆。在连续夺得平安铜鼎、银鼎之后，深入实施“三年平安计划”，有望实现省级平安市创建“十一连冠”。

　　危难之中有真情，灾害面前见精神。我们对突如其来的“11·13”里东山体滑坡灾害大救援记忆犹新，在党中央、国务院亲切关怀，省委、省政府和市委坚强领导下，军地军民心手相连，社会各界大力援助，以最快时间顺利完成了灾害救援，灾后恢复重建正有序推进。“里东大救援”所展现的感人事迹和奉献精神，必将成为推动丽水加快发展的强大正能量！

　　各位代表，过去一年取得的成绩，既为“十二五”发展画好了句号，又使丽水经济社会的发展站在了一个新起点。

　　五年来，生态文明建设在绿色崛起、科学跨越中迈出坚实步伐。“秀山丽水、养生福地、长寿之乡”的区域形象公认度明显提高，生态环境状况指数连续12年保持全省第一、全国领先，生态文明总指数达107.34，居全省第一。我市成为首批国家生态文明先行示范区、第二批全国水生态文明城市建设试点市，创成首批省级生态市。云和、遂昌、庆元、缙云先后创成国家级生态县，国家级、省级生态乡镇比例分别达到53.2%、91.9%，市级生态村比例达到85.8%。综合实力在绿色生态发展中迈上新台阶，地区生产总值从664.76亿元增加到1102.34亿元，年均增长8.9%，人均生产总值达8290美元，各县(市、区)均摘掉欠发达帽子。

　　五年来，产业结构在加快转型、扩量提质中明显优化。三次产业结构从9.5∶49.2∶41.3调整到8.3∶45.6∶46.1。建成35.3万亩粮食生产功能区、89个省级现代农业园区、4条省级农业全产业链，新增市级以上示范农民专业合作社151家、示范家庭农场870家。丽水开发区晋升为国家级经济技术开发区，全市新增国家重点扶持高新技术企业172家、省级科技型中小企业263家，新产品产值率由11.8%提高到33.1%，高新技术产业、战略性新兴产业增加值占规模以上工业增加值的比重分别达到25.6%、18.3%，主导或参与制订、修订国家标准48项、行业标准64项。服务业增加值从274.84亿元增加到507.93亿元，年均增长10.5%，新增9家4A级旅游景区和4个省级旅游度假区、4家省级现代服务业集聚区，旅游总收入相当于GDP的比重从18.1%提高到38.6%，文化及相关产业增加值占GDP比重从2.6%提高到接近5%，成为全国网商发展百强城市和零售网商密度最高的25个城市之一。

　　五年来，城乡面貌在统筹协调、“两美”建设中日新月异。常住人口城市化率从48.4%提高到56.4%，中心城区建成区面积从32.31平方公里扩大到34.90平方公里，“北居中闲南工”发展格局基本形成。成功创建国家园林城市、国家森林城市、国家卫生城市及省级历史文化名城，城市品位大幅提升。通过城乡环境整治，全面消除垃圾河、黑臭河，实现镇级污水处理设施全覆盖，新增污水管网679公里，完成农村生活污水治理1916个村、受益农户36.3万户，行政村自来水普及率100%，77个村进入中国传统村落保护名录，3个村被评为首批中国少数民族特色村寨，6个县(市、区)被评为全省美丽乡村创建先进县(市、区)。“三改一拆”扎实推进，累计拆除违法建筑面积2019.77万平方米，完成“三改”面积2385.96万平方米，完成市区老小区、城中村改造20个。累计完成固定资产投资2817.46亿元，其中重大基础设施投资1103.66亿元。城乡基础设施进一步完善，实现县县通高速、康庄公路村村通和全市公交一卡通，新能源电动汽车分时租赁系统投入运营，迈入“高铁时代”、“4G时代”。

　　五年来，发展活力在改革引领、开放助推中持续增强。全市共争取省级以上改革试点115项，其中国家级38项，推动形成了市域“一盘棋”、县域有特色的经济社会发展格局。莲都成为国家乡村旅游创客示范基地，龙泉成为全国集体林业综合改革试验示范区，云和成为国家中小城市综合改革试点县，青田跻身全国县域经济竞争力和发展潜力“双百强”，遂昌成为全国农村电子商务强县创建先行县，松阳成为全国传统村落保护发展示范县、保护利用试验区，庆元、景宁列入国家主体功能区建设试点示范，景宁跻身全国民族自治县十强，缙云成为省级“腾笼换鸟”综合试点县、清洁能源示范县。农村发展活力全面释放，村经济合作社股份制改革全部完成，农村产权交易累计达1177宗，农村家庭信贷可及率、可得率分别达95.4%、72.4%，比全国分别高出44.2、29.3个百分点。市场主体活力竞相迸发，累计净增市场主体8.94万户，其中私营企业1.37万户，民间投资年均增长24.4%。开放型经济发展水平明显提高，实际利用内资、外资分别是“十一五”时期的3.69倍、3.56倍，外贸出口年均增长18.4%，完成境外投资项目55个，建成3个国家级出口产品质量安全示范区。

　　五年来，民生福祉在共建共享、全面发展中不断呈现新气象。城乡居民人均可支配收入年均分别增长10.3%、13.6%，城乡居民收入比从2.53∶1缩小到2.19∶1。新增就业10.4万人。城乡基本养老、基本医疗保险参保人数分别达158.19万、237.92万，居家养老服务照料中心覆盖70%的城乡社区，建设残疾人庇护中心62家。与全省同步实现义务教育发展基本均衡市目标，学前教育等级园覆盖率从43.1%提高到85.5%。创成全国文化先进县1个、省级文化先进县6个，建成农村文化礼堂317个，基层文化站室实现行政村(社区)全覆盖，跻身中国文化城市20强。公立医疗机构基本药物制度实施率达100%，实现县(市、区)与省级三甲医院合作办医全覆盖。创成省级体育强县5个，国民体质总体合格率达90.6%。深化平安丽水建设，夺得平安银鼎，安全生产事故起数、死亡人数连续12年下降。成功创建全国双拥模范城。民族地区加快发展，少数民族群众人均收入实现倍增。实现市民生活满意度、生态环境质量公众满意度、群众安全感满意率、食品安全群众满意率、卫生状况满意率、社会道德环境公众满意度排名全省第一。

　　与此同时，我们坚定不移推进政府自身建设，认真开展党的群众路线教育实践活动和“三严三实”专题教育，作风建设和反腐败工作进一步加强。切实推进依法行政，市级政府部门权力事项由8342项依法削减为3860项，非行政许可审批事项全面取消，完成规范性文件审核345件，受理行政复议194件，连续6年被评为全省法治政府建设优秀市。加强政府突发公共事件应急处置能力建设，取得“8·20”全流域特大洪灾、“11·13”里东山体滑坡灾害等重大事件应急抢险的胜利。

　　各位代表，“十二五”发展成绩来之不易。这是市委正确领导的结果，是全市人民凝心聚力、团结奋斗的结果。在此，我代表市人民政府，向全市人民以及广大建设者，向人大代表、政协委员，各民主党派、工商联、人民团体和社会各界人士，向驻丽部队、武警官兵和省部属驻丽各单位，向所有关心支持丽水发展的海内外朋友们，表示衷心的感谢！

　　我们也清醒看到，我市经济社会发展还存在不少问题。主要是：面对经济下行和转型升级双重压力，在经济增长从高速向中高速转换过程中，新旧动力转换不畅问题比较突出，产业升级艰难，生态经济发展不够充分，地区生产总值、工业增加值等部分经济指标未能实现2015年和“十二五”预期增速；城乡居民尤其是已脱贫的低收入农户持续增收基础还不稳固，民生实事的谋划与群众需求还有落差；防灾减灾、安全生产、企业风险化解等领域潜在的问题还有不少，维护社会和谐稳定面临新的挑战。在政府自身建设方面，政府职能转变、依法行政、行政效能与群众期望还有差距，一些工作人员懒政庸政怠政现象依然存在，消极腐败现象仍有发生。我们一定高度重视，用心用力解决好这些问题。

　　二、“十三五”时期的奋斗目标和主要任务

　　“十三五”是丽水拓展生态文明发展空间，与全省同步高水平全面建成小康社会决战决胜的五年。根据中共丽水市委《关于制定丽水市国民经济和社会发展第十三个五年规划的建议》，市政府编制了《丽水市国民经济和社会发展第十三个五年规划纲要(草案)》，提请大会审议。经本次大会批准后，市政府将认真组织实施。

　　“十三五”发展的指导思想是：高举中国特色社会主义伟大旗帜，以马克思列宁主义、毛泽东思想、邓小平理论、“三个代表”重要思想、科学发展观为指导，深入贯彻习近平总书记系列重要讲话精神，以“四个全面”战略布局为统领，以创新、协调、绿色、开放、共享五大发展理念为引领，以省委“八八战略”为总纲，坚持“绿水青山就是金山银山”战略指导思想，坚持生态环境保护与生态经济发展并重，完善生态文明体制机制和发展方式，协同推进新型工业化、城镇化、信息化、农业现代化和绿色化，着力实施“151”绿色发展行动计划，推进“绿色发展、生态富民、科学赶超”，打造“两山”样板，争当“双区”示范，加快建设美丽幸福新丽水，与全省同步高水平全面建成小康社会。

　　“十三五”发展的主要目标是，确保实现已经确定的“四翻番”目标，与全省同步高水平全面建成小康社会。全市生产总值年均增长7.5%左右，城乡居民人均可支配收入年均分别增长7.5%、10%。完成“151”绿色发展行动计划，即生态环境状况指数继续保持全省第一、全国领先，五年累计完成固定资产投资5000亿元，人均生产总值超过1万美元，并使社会保障覆盖率达到100%、全社会研发经费支出占生产总值比重达到2%左右，努力实现生态经济更加发达、生态环境更加优美、设施网络更加完善、人民生活更加美好、制度建设更加成熟。

　　实现上述目标，必须全面贯彻五大发展理念，落实好事关丽水长远发展的重大工程、重大项目、重大政策，集中力量抓好既该干又能干成的大事。

　　(一)贯彻创新发展理念，增强生态文明建设新动力

　　深化改革创新，释放改革红利。深入推进以生态文明先行示范区建设为龙头的国家级改革试点，积极争取改革扩面，重点争创全国旅游业改革创新先行区、农村电子商务建设先行示范区、深化集体林权制度改革综合试验示范区。推进县域经济综合改革，完善“亩产效益”和生态效益并重的资源要素市场化配置机制，强化节能节地节水、环境、技术、质量、安全等市场准入标准，力争2020年覆盖到所有新建项目和存量企业。着眼于激发金融、社会、国有资本活力，在基本完成农村产权制度改革的同时，继续深化农村金融改革，加快构建普惠型农村金融体系；大力发展科技金融和绿色金融，积极争取民营银行试点；成立基础设施投资基金，鼓励社会资本以独资、PPP等形式进入基础设施、社会事业领域；加快国资公司转型发展，推进“水电入市”。着眼于激发市场主体活力，综合推进“四张清单一张网”、审批、商事制度改革，力争到2020年全面实行行政管理和公共服务“一站式”在线运行，完善负面清单，鼓励众创、众包、众扶、众筹机制创新。

　　全力培育“195”生态产业体系。“1”就是围绕到“十三五”期末把生态旅游产业培育为千亿级的第一战略支柱产业，全面推进国家全域旅游示范区、省级旅游综合改革试验区建设，加快旅游公司实体化、旅游景区资本化、旅游资产证券化改革，促进以国家5A级景区和国家级旅游度假区创建为重点、高等级旅游产品为平台的景区转型提升，争创国家公园，努力成为浙皖闽赣国家东部生态文明旅游区的主角。“9”就是积极发展高端装备制造、节能环保、生物医药、新材料、信息经济、绿色能源、健康、文化、生态精品农业等9大产业。“5”就是把民宿、机器人制造、电子商务、时尚革制品、资源精加工产业培育为5大新增长点。要以市场化为导向、以“互联网+”为依托，以实施生态精品农业“912”工程、“生态制造2025”、生态服务业集聚区建设计划和发展总部经济等为载体，大力推进产业融合发展。“十三五”期间，力争建成5条综合产值超100亿元的农业全产业链，战略性新兴产业、装备制造业、高新技术产业增加值占规上工业的比重和园区集中度、企业集中度年均分别提高0.5个百分点。

　　加快打造创业创新平台。建设“一核一带多点”产业主平台，以丽水生态产业集聚区为核心，打造生态工业发展平台；以瓯江干流及主要支流为纽带，打造瓯江生态经济走廊；全力推进30个以上省、市级特色小镇建设。加快完善创新体系和创新平台，高水平打造绿谷信息产业园、物联网产业园、丽缙五金科技产业园、丽景民族工业园，建设一批创新苗圃和孵化基地，积极推进国家知识产权试点城市建设，到“十三五”期末建成省级创新型城市。推进地校战略合作、科技大市场建设，力争每个优势产业建成一个公共创新服务平台。依托“三园一地”等平台，加强高层次、高技能、紧缺急需和地方特色人才队伍建设，打造人才生态最优城市。到2020年，力争全市人才资源占人力资源比重达到26%。

　　(二)贯彻协调发展理念，推动市域统筹均衡优化发展

　　强化中心城市核心带动功能，加快推进新型城市化。围绕更好地顺应“高铁时代”，加快建设生态旅游名城。进一步完善中心城区“北居中闲南工”的功能布局，突出“中闲”区块，即环南明湖区块休闲养生度假旅游产业培育和设施配套，统筹推进城东区块及城西、水南、江滨开发建设，加快联城区块配套基础设施建设，打造山水生态城市新标杆。拓展城市发展空间，构建“中心城区+4个卫星城区”发展格局，促进形成“20分钟交通圈”；把云和、松阳纳入中心城市未来发展空间统一规划研究。以省部共建城乡建设转型综合示范区为抓手，加快推进户籍制度改革，促进农业人口转移，实现山区农民异地转移5万人，“十三五”末常住人口城市化率达到70%。以创建全国文明城市、打造“智慧城市”和实现国家卫生县城全覆盖等为抓手，不断提升城市品位。

　　以构建“四区四级两轴”城乡空间发展新格局和“四美”城乡建设为推动，扎实推进城乡一体化。以主体功能区规划为指导推进分区管制化、城镇组团化、功能一体化，把市域发展划分为城市核心区、城市拓展区、生态经济区、生态功能区四大分区，促进“美丽丽水、美丽县城、美丽小镇、美丽乡村”四级开发，形成1个中心城市组团、10个小城市组团、20个左右中心镇、200个以上美丽乡村的“1122”城乡体系。依托高速公路、高速铁路，串联东西、南北两个方向的城镇发展轴，加快区域一体化进程。推进“四美”城乡建设，加强历史文化街区、历史文化村落保护利用和“处州民居”、“花样村庄”建设，促进美丽城乡和美丽经济联动发展。

　　推动民族地区加快发展。加大对景宁畲族自治县和其他民族乡(镇)、民族村的政策扶持力度，积极推动民族地区全面小康建设。通过支持景宁创建民族地区可持续发展改革试点、发展民族总部经济、建设全国畲族文化发展基地等举措，促进景宁发展走在全国民族自治县前列。

　　(三)贯彻绿色发展理念，打造“秀山丽水”生态典范

　　严格落实生态红线管控，完善生态绩效考核。贯彻落实“811”美丽浙江建设行动方案，全面实施环境功能区划，加强环境监管网格化管理，推进环保机构监测监察执法制度创新。实施绿色GDP核算和考核，到2017年，生态文明建设指标占党政绩效考核比重达45%以上。

　　强化生态治理和生态保护。统筹推进治水治气、治城治乡、治土治山，“三改一拆”、“五水共治”等转型升级组合拳要贯穿整个“十三五”，决不把违法建筑、污泥浊水、脏乱差的环境带入全面小康，确保实现省级生态文明建设示范区全覆盖。到2020年，实现农村生活污水处理全覆盖，市区污水收集处理率达到95%、县级以上城市污水集中处理率达到90%，全市Ⅰ-Ⅲ类水质断面比例保持在98%以上，消除劣V类地表水；全市环境空气质量均达到二级标准要求，环境空气质量指数(AQI)优良天数比率保持在90%以上；县县实现“清三河”达标、环境空气质量优良、基本无违建，有效控制农业“两区”土壤污染，基本消除重大地质灾害隐患；森林覆盖率保持在80%以上，实施美丽林相改造与建设320万亩。

　　推进绿色低碳循环发展。深入实施节能降耗“双控工程”，推进园区生态化改造，大力发展生态产业和节能环保产业，工业危险废物实现无害化处理。全市公交车、出租车、营运专车、一般公务用车替换为新能源汽车或改造为清洁能源汽车。

　　(四)贯彻开放发展理念，以大开放促进大发展

　　以“五网”建设夯实开放基础。按照智慧互联的要求，统筹推进综合交通、生态保护、绿色能源、智慧信息、社会服务五大基础网络建设。围绕打造支撑美丽经济发展的交通走廊，大力推进新一轮交通大投入、大建设、大发展。以向“县县通铁路”迈进为目标，重点建设衢宁、衢丽、金台铁路，积极推动温武铁路前期研究，推进形成“两纵两横”铁路干线布局。完善“两横两纵三支”公路网，加强通景公路、县际通道建设，有序推进国省道、农村公路改造提升，力争武松龙、遂江上、庆寿等高等级公路纳入上级规划。加快“一干三支两港”港航建设，建成丽水至青田高等级航道。谋划“112X”航空网，力争建成丽水机场、迈入“航空时代”。切实抓好环境基础设施“12310”工程建设。推进大溪治理、丽水盆地易涝区防洪排涝、中小河流治理等水利工程建设，实现中心城区和主要区域全面达到20—50年一遇防洪标准。积极打造华东绿色能源基地，加快园区集中供热设施建设，实施管输天然气“县县通”工程。进一步完善以1000千伏为骨干、500千伏为支撑，各级电网协调、城乡供电可靠的智能电网。建设智慧政务、智慧旅游、智慧交通等智慧应用体系，实现县级以上公共区域场所及A级以上景区免费Wi-Fi全覆盖。推进一批教育、文化、卫生、体育、养生(养老)、便民服务项目建设，努力满足各类民生服务需求。

　　对接国家“一带一路”倡议，提高外向型经济发展水平。积极推动贸关检税汇一体的大通关建设，建设跨境电子商务产业园。支持青田培育华侨总部经济、建设侨乡进口商品城，支持龙泉建设世界瓷都和历史经典文化小城。推进精准招商，力争“十三五”期间累计实际利用内资2000亿元、外资10亿美元。扩大与长三角、海西、珠三角经济区的交流合作，加强“中国长寿之乡”、“中国气候养生之乡”等品牌的推广宣传，促进区域协作和融合。

　　(五)贯彻共享发展理念，不断增强人民群众幸福感

　　促进城乡居民普遍持续增收。坚持就业优先战略，完善收入分配制度和劳动报酬增长机制，推进“无欠薪丽水”建设。全力促进农民增收，加强低收入群体增收帮扶。到2020年，新增城镇就业7万人，实现零就业家庭动态清零，确保城乡居民人均可支配收入比2010年翻一番。

　　以标准化推动公共服务均等化。高标准普及15年基础教育，到2020年，义务教育标准化学校比例达到90%以上，等级幼儿园、普惠性幼儿园比例分别达到90%、80%以上，力争与全省同步实现教育现代化。促进丽水学院向应用型大学发展，支持丽水职业技术学院打造全国知名的现代高职教育品牌，支持丽水广播电视大学向高水平现代化远程开放大学转型。大力实施全民参保计划，完善养老、扶残等服务体系，以及适度普惠型儿童福利保障体系、城乡住房保障体系。深化医疗、医保、医药“三医联动”改革，大力发展全民健身，建设“健康丽水”。加快发展中医药和畲医药事业。实施一对夫妇可生育两个孩子政策，统筹做好妇女儿童、老龄、残疾人等工作，加强流动人口服务管理。关注失独老人，关爱留守儿童。繁荣“瓯江文化”，实施基本公共文化服务标准化均等化五年行动计划，建成“城市15分钟、农村30分钟”文化活动圈。

　　不断提升现代化治理能力。深化平安丽水、法治丽水建设，全面推行平安报表管理法，实行社会风险隐患清单式管理，完善基层社会治理网络体系和矛盾纠纷大调解机制、阳光信访和法治信访机制，推进社会信用体系建设，健全防灾减灾、安全生产、食品安全、应急联动等社会公共安全体系，强化社会治安立体防控体系，努力打造人民群众最有安全感的城市。积极推进工青妇等群团工作及慈善、移民、档案等事业新发展，进一步抓好民族宗教、侨台外事工作。推进军民融合深度发展，做好国防建设、国防教育、国防动员、人民防空和双拥优抚安置工作。

　　三、2016年主要工作安排

　　2016年是“十三五”开局之年。我们所面对的发展形势，既有不确定性、不稳定性和经济下行压力，更有对新常态适应性、引领性不断增强的一面。我们所面临的发展环境，既富有挑战，更充满机遇，尤其是随着省委省政府支持丽水建设全域生态功能区，“绿水青山”转化为“金山银山”的空间将越来越广阔，红利将越来越显现。站在历史的新起点，我们必须坚定发展自信、保持战略定力，正视发展困难、勇于面对挑战。

　　综合分析各方面因素，建议今年全市经济社会发展的主要预期目标为：地区生产总值增长7-7.5%；规模以上工业增加值增长6%左右；服务业增加值增长8%以上；固定资产投资增长10%以上；社会消费品零售总额增长10%左右；外贸出口保持适度增长；一般公共预算收入增长7%；旅游总收入增长25%；城镇居民人均可支配收入增长7%以上，农村居民人均可支配收入力争增长10%；城镇新增就业岗位1.4万个，城镇登记失业率控制在4%以内；治水治气、节能减排完成省下达目标任务。

　　开局决定全局，起跑决定后势。提出并努力实现以上预期目标，既是“十三五”发展开好局、起好步的内在要求，也是面对错综复杂的经济形势、严峻的经济下行压力的自我加压、自我挑战。我们必须认真贯彻中央和省委、市委决策部署，坚持“绿水青山就是金山银山”战略指导思想，以归零的心态、翻篇的姿态、奋发的状态和看齐的意识，变压力为动力，在推进“绿色发展、生态富民、科学赶超”，打造“两山”样板、争当“双区”示范的道路上迈出新步伐。必须以“严”、“实”的要求，集中精力找短板、全力以赴补短板，坚决守护好全省生态屏障，加快壮大生态经济，加强供给侧结构性改革，厚植发展优势，增强发展动力，不断凸显生态底色、发展特色、民生本色、担当气色，加快与全省同步高水平全面建成小康社会步伐。

　　(一)努力实现创新制度供给的良好开局

　　深化生态文明制度创新，增强可复制可推广性。全力推进“生态+”行动，开展编制自然资源资产负债表省级试点，探索建立生态文明的“丽水标准”，组织开展“丽水生态文明日”系列活动，推进环境监管网格化管理全覆盖，认真实施生态环境损害责任追究办法，做好市容与环境卫生管理等方面生态立法的基础工作。继续推进各级各类改革试点，不断扩大改革效果。

　　加大重要领域和关键环节改革力度。针对经济转型升级，加快去产能、去库存、去杠杆、降成本、补短板相关改革，建立健全优胜劣汰机制，依法依规处置“僵尸企业”、严厉打击“逃废债”行为，继续关停落后产能，整治“低小散”企业；在市长热线、市长信箱开通服务企业专栏，开展“精准帮扶企业促发展”专项行动，建立政银企对接、企业发展情况分析常态化机制，及时解决影响企业发展的突出问题，继续为亿元以上企业、高成长型企业提供“一对一”服务措施，当好服务市场主体的“店小二”，构建“亲”、“清”新型政商关系；制定实施企业减负三年行动计划，切实降低实体经济企业成本；强化资源要素市场化配置，大力支持企业主动对接多层次资本市场，加快生态经济产业基金市场化运作，增强产业发展动力。针对进一步激发农村发展活力，结合加快不动产统一登记，联动推进农村产权、金融改革；加快构建“三位一体”农民合作经济组织体系。针对投融资难，积极探索政府资产证券化、政府投资基金、政府投资公司等多元化投融资体制和资本运作机制，在财力允许前提下，大力推广PPP模式，加快市农发公司、旅投公司等国资营运机构市场化转型。

　　(二)努力实现生态经济发展的良好开局

　　着力推进生态经济化、经济生态化。坚持在发展中保护、在保护中发展，把握供给侧结构性改革机遇，紧扣人民群众对清新空气、干净饮水、安全食品、优美环境的紧缺需求，加强稀缺生态产品的有效供给，把生态环境优势充分转化为经济优势、竞争优势和发展优势。发起成立并依托“中国长寿之乡绿色产业联盟”、“中国(长三角)高铁旅游联盟”，提升丽水生态经济影响力。加强以交通为重点的基础设施规划与统筹，促进生态经济发展。

　　着力打造生态产业升级版。坚持把生态农业作为富民增收的基础产业，组织实施生态精品农业“912”工程，新加入使用“丽水山耕”品牌农产品100个以上，以农业品牌化、电商化巩固扩大生态化、标准化，实现市级以上农业龙头企业质量安全可追溯体系全覆盖、网上农产品营销普及率达70%；把打造美丽田园与发展休闲、创意农业有机结合起来，启动10个省级现代农业综合区景区化提升项目，新建休闲养生观光农业区点20个；启动农业产业集聚区和农业特色强镇建设，推广“一亩山万元钱”林下经济发展模式。坚持把生态工业作为产业转型升级的突破口，统筹抓好新兴产业培育和传统产业改造提升，制定发布丽水“生态制造2025”实施方案，深化“四换三名”、企业技改“双百工程”，实施100个市级以上战略性新兴产业项目，工业技改投资增长12%以上；加快机器人产业集聚发展，力争引进国内机器人骨干企业；积极推进园区整合及循环化改造试点，促进企业兼并重组；完善涉工部门服务开发区发展长效机制，合力助推开发区打造生态工业主阵地。坚持把生态旅游业作为第一战略支柱产业和现代服务业的龙头来抓，以旅游“三化”改革为引擎，不断加大旅游投资、创新旅游产品供给，确保旅游投资增长15%以上；加快以“中闲”区域为核心的中心城市旅游产品开发，扎实推进古堰画乡、遂昌金矿、缙云仙都、青田石门洞等景区创5A工作，积极谋划龙泉山、云和梯田、千峡湖5A景区创建，力争古堰画乡景区列入国家5A级景区培育名单；加快实施乡村旅游转型升级发展三年行动计划，打响“丽水山居”民宿品牌，新创建30个乡村特色民宿示范乡镇、村、点。加快发展生产性和生活性服务业，加快发展文化创意、历史经典产业，促进建筑业转型升级和房地产业健康发展。

　　着力强化创新驱动。加快打造创新平台，全力抓好特色小镇建设，争取完成投资180亿元，支持各类改革试点在特色小镇先行先试，按照每个特色小镇都要建成3A级以上景区的要求，重点推进示范小镇建设；切实抓好丽水开发区机器人、新材料产业孵化器及生物医药产业加速器建设，每个县(市、区)年内至少建成一个使用面积不低于3000平方米的创业孵化基地，力争丽缙五金科技产业园创成省级高新技术产业园区。加快培育创新主体，力争新增国家重点扶持的高新技术企业、省级科技型中小企业和省级农业科技企业共110家以上，省级及以上研发机构10家以上。统筹抓好产业融合和产业创新，在工业化和信息化深度融合、制造业服务化，特别是农旅融合、文旅结合等方面下功夫，提升产业竞争力；联动推进“互联网+”、“机器人+”、“标准化+”行动计划，推进标准强市、质量强市、品牌强市。

　　(三)努力实现扩大有效投资的良好开局

　　保持有效投资力度，推进供给端和需求端协同发力。加快衢宁铁路、缙云抽水蓄能、千峡湖旅游度假区3个百亿级项目建设，力争开工建设丽水机场、金台铁路丽水段、松阳黄南水库、好溪水利枢纽等一批10亿级项目，积极推进丽水高铁新站房等项目建设，建成龙浦高速公路，谋划和推出更多PPP项目，确保固定资产投资增长10%以上。更加注重结构优化，确保重大基础设施、重大产业项目、高新技术产业、生态环保投资较快增长。发挥华侨优势，积极推进跨境电子商务出口、名优特产品出口。大力培育旅游、文化、信息、健康、养生养老等新的消费热点，积极推广“赶街”模式，促进网络市场健康发展，网络零售额增长35%左右。

　　深化“五资齐抓”，推进精准招商。强化招商引资“一号工程”主体责任，建立招商项目市、县两级领导干部全员、全程、全责任联系制度和招商项目落地“三色预警机制”，完善招商考核办法。力争全年实际利用内资295亿元、外资2亿美元，浙商回归到位资金145亿元，引进大项目100个；签约“山海协作”项目100个，到位资金140亿元。

　　(四)努力实现美丽城乡建设的良好开局

　　以精美规划引领城乡建设。强化“山水林田湖”生命共同体理念，推进“多规合一”改革及新一轮城乡规划体系建设，强化城市风貌控制，提升城市形态、园林景观、城市小品、城市建筑设计水平，加强沿线沿景村庄规划编制和实施。按照功能定位、历史传承和文化积淀有机统一的原则，防止规划设计“千城一面”、“千村一面”。

　　以精致建设推动城乡发展。推动中心城区配套基础设施项目围绕高铁站、港口、机场等重要节点科学布局，开工建设高铁站周边道路、好溪路二期等城市道路，抓好高铁站周边区块环境改造，推进中山街等区块地下管网循环系统升级换代和天宁等区块城市有机更新。继续抓好市区老小区、城中村改造，深化城市“六个一”示范工程，推进“坡地村镇”建设用地试点，推进“海绵城市”建设。全市新增城市道路30公里、城镇污水管网90公里，新增绿道100公里、城市公园绿地30公顷，新开工地下空间40万平方米，新增停车位3000个。大力推进“五水共治”，努力夺取“大禹鼎”。协同推进“三改一拆”、“六边三化三美”和城镇危旧住宅房屋治理改造，建成15个省级美丽宜居示范村，改造提升10条美丽乡村风景线。争创2个基本无违建县(市、区)，60%以上乡镇(街道)建成无违建乡镇(街道)。实施城镇低效用地再开发1320亩，盘活存量建设用地3300亩。

　　以精细管理提升城乡品质。全面推进全国文明城市创建工作。推进“智慧城市”建设，完善市、区共建机制，强化“数字城管”和综合行政执法，理顺高铁站区域综合管理体制。启动7个县(市)的国家卫生县城创建工作。深化户籍管理制度改革，全面实施居住证制度，促进有能力在城镇稳定就业和生活的农村人口进城落户。

　　(五)努力实现民生事业发展的良好开局

　　抓好就业增收和社会保障。加强创业培训和就业指导，促进高校毕业生就业创业，做好农民转移就业服务和城镇就业困难人员帮扶工作，提高就业服务的精准性。着力巩固消除4600元以下低收入农户成果，在精准帮扶、防止返贫上下功夫，重点帮助低收入人群增强自我发展能力。完成第二轮特扶项目建设，抓好农民增收“老三宝”和“新三宝”，推进高山、远山和地质灾害隐患区农户异地转移，启动城区新安置区块建设。健全大社保体系，推进全国工伤预防试点城市创建，完善城乡最低生活保障和社会救助制度，落实重度和困难残疾人护理与生活补贴政策。

　　抓好各项社会事业发展。深化社会事业综合改革，抓好各类事业单位统一登记管理试点，鼓励和引导社会力量兴办社会事业。加强推进教育现代化、教师队伍建设的制度供给，加快学校项目建设，深化基础教育课程改革，全面实施学前教育第二轮三年行动计划和特殊教育三年提升计划，不断提高育人水平。深化公立医院综合改革，深入推进“双下沉、两提升”工作，加强乡、村卫生院(室)建设，稳步推进分级诊疗制度，完成市第二医院搬迁工程并投入使用。大力发展养老服务，全面实施养老服务补贴制度，推进农村敬老院改造转型和居家养老服务照料中心多模式运营，新增养老机构床位2000张，新建居家养老服务照料中心280个。推进“丽水乡村春晚”创建国家公共文化服务体系示范项目，打造100台特色乡村春晚。以中英联合纪念汤显祖、莎士比亚逝世400周年为契机，把汤显祖文化打造为国家对外交流重要品牌。市体育馆投入使用。办好亚洲轮滑锦标赛、中华龙舟赛、丽水马拉松等重要赛事，积极培育发展赛事经济。

　　抓好“平安丽水”建设。打好“三年平安计划”决胜战，全力夺取平安金鼎。全面开展“七五”普法。抓好防灾减灾和反恐防暴、信访维稳、安全生产、食品药品安全、公共安全等各项工作，加快构建立体化社会治安防控体系，推进食品安全溯源体系建设，加强互联网综合治理和“扫黄打非”工作。优化金融生态环境，维护区域金融稳定。全面加强地质灾害隐患排查治理，严格落实地质灾害防治责任。加强国防动员工作，支持驻丽部队建设，深化军民融合发展。全力做好G20峰会相关工作。

　　抓好民生实事。继续加大民生投入，确保民生支出占财政支出的三分之二以上。在确保完成省里部署的民生实事的同时，今年我们按照民生实事“群众提、群众定”的理念和办法，确定了十个方面实事项目。市政府将健全工作机制，明确任务表、时间表、责任表，切实办好这些民生实事。具体包括：丰富城乡文体生活、强化食品安全治理、努力促进就业创业、持续改善交通出行、加强大气治理力度、推进教育惠民工程、全力建设美丽城乡、积极发展农民增收“新三宝”、进一步提升社会服务水平、加大水环境治理力度。

　　(六)努力实现政府自身建设的良好开局

　　着力建设实干政府。政府工作必须干字当头冲在前，苦干实干争上游，善作善成创实绩。要勇于担当，以逢山开路的闯劲、虎口夺食的拼劲、久久为功的韧劲，干一件成一件，努力干出一个好的开局。围绕一种声音落地、一个目标奋斗、一个态度落实、一张蓝图干到底，下苦功夫加强政风建设。抓工作要具体化、项目化、责任化，要挂出作战图、制定项目书、设定目标值、倒排时间表、落实责任制，决不允许决策不决断、说话不干活、人浮于事、推诿扯皮、“职责真空”、遇到矛盾绕着走。

　　着力建设法治政府。政府工作必须全面纳入法治轨道，要坚持依法行政，继续深化“四张清单一张网”改革，切实做到法定职责必须为、法无授权不可为，不断提高公信力。决策和用权决不能随意任性，必须讲法定程序、法律责任，提交讨论的事项必须经合法性审查。解决问题要增强法治意识、规则意识，带头维护社会公平正义。

　　着力建设廉洁政府。政府工作必须坚持清正清廉清明，对自身要求要严之又严。要深化“三严三实”专题教育和“四风”整治，坚决整治懒政庸政怠政，坚持有案必查、有责必问。要带头树立崇俭戒奢的文明新风，严控“三公”经费和会议费，全面完成公务用车改革。要把自觉接受人大监督、政协民主监督、司法监督、审计监督和社会监督、舆论监督作为廉洁自律新常态，兢兢业业，干净干事干成事。

　　各位代表，“十三五”发展蓝图十分宏伟，前景十分美好。让我们更加紧密地团结在以习近平同志为总书记的党中央周围，在省委、省政府和市委的坚强领导下，归零翻篇开新局，精准发力补短板，为加快推进“绿色发展、生态富民、科学赶超”，建设美丽幸福新丽水，与全省同步高水平全面建成小康社会而努力奋斗！
